# Recent Progress in Isotropic Magnetorheological Elastomers and Their Properties: A Review

**DOI:** 10.3390/polym12123023

**Published:** 2020-12-17

**Authors:** Muhammad Arslan Hafeez, Muhammad Usman, Malik Adeel Umer, Asad Hanif

**Affiliations:** 1 School of Civil and Environmental Engineering, National University of Sciences and Technology, Islamabad 44000, Pakistan; muhammad_arslanhafeez@yahoo.com; 2 School of Chemical and Materials Engineering, National University of Sciences and Technology, Islamabad 44000, Pakistan; umer.adeel@scme.nust.edu.pk; 3 Institute of Applied Physics and Materials Engineering, University of Macau, Avenida da Universidade, Taipa, Macau SAR, China

**Keywords:** magneto-sensitive smart materials, magnetorheological elastomers (MRE), carbonyl iron particles, rheological properties, construction and medical applications

## Abstract

Magnetorheological elastomers (MREs) are magneto-sensitive smart materials, widely used in various applications, i.e., construction, automotive, electrics, electronics, medical, minimally invasive surgery, and robotics. Such a wide field of applications is due to their superior properties, including morphological, dynamic mechanical, magnetorheological, thermal, friction and wear, and complex torsional properties. The objective of this review is to provide a comprehensive review of the recent progress in isotropic MREs, with the main focus on their properties. We first present the background and introduction of the isotropic MREs. Then, the preparation of filler particles, fabrication methods of isotropic MREs, and key parameters of the fabrication process—including types of polymer matrices and filler particles, filler particles size and volume fraction, additives, curing time/temperature, and magnetic field strength—are discussed in a separate section. Additionally, the properties of various isotropic MREs, under specific magnetic field strength and tensile, compressive, or shear loading conditions, are reviewed in detail. The current review concludes with a summary of the properties of isotropic MREs, highlights unexplored research areas in isotropic MREs, and provides an outlook of the future opportunities of this innovative field.

## 1. Introduction

The materials, i.e., piezoelectric [1,2], biomimetic [3], thermochromic [4], electrorheological [5], thermoelectric [6], photochromic [7], magneto-sensitive [8], magneto-active [9,10], and shape memory alloys [11], intelligently respond to variations in the surrounding conditions and are termed as smart materials [12]. The magnetorheological (MR) materials, invented by Jacob Rabinow in 1948, are magneto-sensitive smart materials [13,14]. These materials are produced in different forms, such as MR foams, MR elastomer (MRE), MR gel [15], and MR fluid (MRF) [16,17,18]. Although the response time of MRE is slower than MRF [19], MRE still effectively overcomes the deficiencies of MRF, particularly the particle sedimentation, leakage, and environmental contamination problems [20]. Due to their rapidly and reversibly controllable properties, including morphological [21], tomographical [22], mechanical [23], dynamic mechanical [24,25,26], magneto-mechanical [27,28], magneto-shear [29], rheological and melt rheological [30], complex torsional [31], physicochemical [32], thermal [33], friction and wear [34], fatigue life [35], and viscoelastic [12] properties, as well as a fail-safe feature [19], MREs have a wide range of applications. These applications include damping and smart sensing in vibration absorbers [36,37] and vibration isolators [38,39,40,41], other sensing devices [42,43], engine mounts, vehicle seat suspension [44], adaptive stiffness devices, actuators to control the flow [31], MR elastic polishing composites [45], seismic dampers and base isolators [46,47,48,49,50], multilayer MRE-based vibration isolators [51], MREs embedded beams [52], variable impedance surfaces, artificial muscles [24], deformable wings [53], MRE embedded sandwich plates [54], adaptive blades [55], active vibration isolation platforms [56,57,58], tunable absorption systems [59], MREs and MRFs based isolators [60], and dielectrics for plane capacitors [61,62]. MREs have also been used in soft, small-scale continuum robots with navigation and active steering capabilities [63]. The polydimethylsiloxane (PDMS)-based sterilizable and biocompatible MREs have recently been introduced for medical and cellular intervention. Moreover, MREs are the most suited candidates for minimally invasive surgery (MIS) and robotic MIS (RMIS) applications [64]. Several numerical studies and models investigating the performance of MREs have also been developed [65,66,67,68].

MREs are generally fabricated from three thoroughly mixed primary components, including elastomeric material (matrix), magnetic filler particles, and additives [69]. The structure of MREs consists of micro- to nanosized filler magnetic particles, dispersed in a polymeric nonmagnetic matrix [70,71,72]. MREs have been fabricated from various types of matrix materials, such as natural rubber, polyurethane (PUR) rubber [73], silicone rubber (SR) [74], ethylene propylene diene rubber (EPDM) [75], and PDMS rubber [76]. Similarly, a variety of magnetic filler particles have been utilized in fabricating MREs, but bare iron particles (BIPs) [77] and carbonyl iron particles (CIPs) [78] are the most widely used magnetic filler particles. These particles are used in different shapes (sphere, flower, flake, and nugget) and sizes (5 to 100 μm). It has been reported that CIPs with an average diameter of 1–9 μm in the volume concentration from 25% to 30% offer ideal magnetic filler particle properties [31]. To further improve the properties of MREs, several additives, including plasticizers, silane coupling agents, and nanosized particles—such as carbon black, carbon nanotubes, graphite, and graphene—have also been incorporated [61,79]. Plasticizers improve elastomer mobility, matrix/filler affinity, and reduce the viscosity of matrix and storage modulus [80]. Silane coupling agents modify the surface properties of filler particles and improve their compatibility with the matrix [13]. Similarly, the addition of nanosized particles, particularly carbon black powder, increases the MR effects and tensile strength and decreases the damping ratio of MREs [24].

MREs can be classified into two groups based on the application of the magnetic field during vulcanization [81]. The MREs, cured without the application of the magnetic field and possessing uniformly distributed filler magnetic particles in the elastomeric matrix, are termed as isotropic MREs [82]. On the other hand, MREs cured in the presence of an external magnetic field and possessing a chainlike columnar structure with filler magnetic particles aligned along the applied magnetic field direction are known as anisotropic MREs [83]. Although the isotropic MREs provide smaller MR effects and relatively slow time response to the externally applied magnetic field than anisotropic MREs, their fabrication is much simpler and easier than anisotropic MREs. This is because the fabrication of anisotropic MREs needs a significantly high magnetic field strength (0.8 T) during crosslinking [24]. Additionally, upgraded rubber processing instruments and a properly designed setup to successfully apply the magnetic field are required to fabricate anisotropic MREs with improved properties. Furthermore, thicker anisotropic MREs cannot be fabricated, because an increase in the thickness of MRE rapidly decreases the magnetic flux density [24,84]. Isotropic MREs provide significant properties for a wide range of industrial applications at a low cost compared to anisotropic MREs. Due to these advantages, isotropic MREs are achieving great industrial importance nowadays [85]. Depending upon the applied magnetic field strength, the moduli of MREs immediately alter due to strong magnetic forces between magnetic filler particles. The ratio of change in moduli with an applied magnetic field to the initial modulus is called the MR effect [77,86]. Till now, different MR effects ranging from 4% to as high as 24,515% have been achieved [19]. Although the functionality, MR effect, and abrupt time response to the applied magnetic field of anisotropic MREs are much better than isotropic MREs, their fabrication is quite difficult compared to isotropic MREs [87].

The major objective of this work is to review the recent progress in isotropic MREs with the main focus on their properties. After the introduction (Section 1), fabrication of isotropic MREs including preparation of magnetic filler particles, fabrication methods, process parameters, types of elastomeric matrix materials and magnetic filler particles, and additives will be reviewed in Section 2. Section 3 will provide details about the properties of new and conventional isotropic MREs and the effects of various factors, including types of elastomeric matrix, magnetic filler particle, and additives; temperature, fraction, and size of magnetic filler particles; the viscosity of matrix/filler mixture before curing; strain frequency, strain amplitude, magnetic field strength, and types of loading conditions on the properties of isotropic MREs. A few highlights of this review, unexplored areas of isotropic MREs, and future recommendations will also be proposed in Section 5. The rapidly increasing use of MREs in various applications is evident in the literature. This review will provide a collection of information on recently developed and conventional isotropic MREs to the concerned researchers, industries, and end-users in this field.

## 2. Fabrication of Isotropic Magnetorheological Elastomers

**Figure 1:**
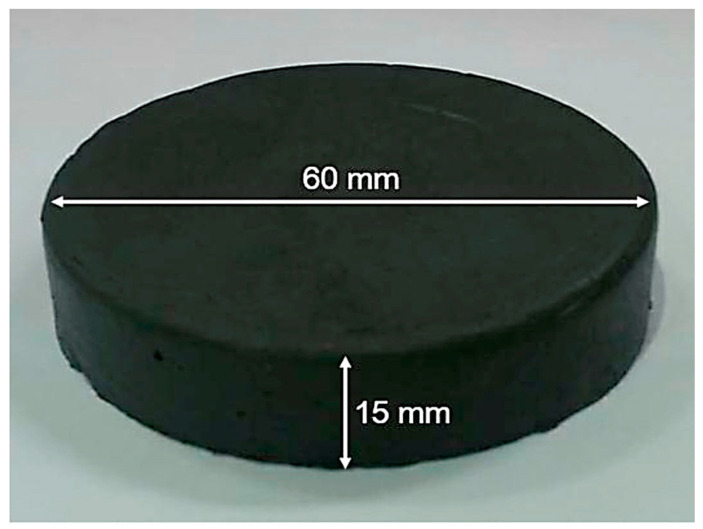
Fabricated isotropic magnetorheological elastomer (MRE) sample and its dimensions [34] (reprinted with permission from Elsevier^TM^).

MREs are composed of three major constituents: an elastomeric matrix, magnetic reinforcing particles, and additives. A variety of elastomeric materials, including liquid silicone [73], room temperature vulcanized (RTV)-based SR [31,45], high temperature vulcanized (HTV)-based SR [88], EPDM rubber [24], PUR [12,70], PDMS rubber [77,81], propylene rubber [30], SR resin [89], natural rubber (NR) [84], and scrub tire rubber [33] have been used for the fabrication of MREs. Among all, SR is the most extensively used rubber due to its unique properties such as room temperature vulcanization, ease of handling and processing, a wide range of operating temperatures, excellent hardness, stiffness [31], nontoxicity, and aging resistance [34]. Various magnetic reinforcing particles, i.e., BIPs [81,90], CIPs [21,69,91], Penta CIPs [92], magnetite (Fe_3_O_4_) [33], titanium dioxide (TiO_2_) [61], and hard FeNdB [93,94] particles have been utilized for the fabrication of MREs. However, CIPs are the most commonly employed magnetic particles due to their high saturation magnetization and a wide range of particle size availability (1–200 μm) [95]. A fabricated MRE sample is illustrated in Figure 1, whereas various types of elastomeric matrices, magnetic filler particles, additives, and key parameters—used in the fabrication of isotropic MREs—are tabulated in Table 1.

### 2.1. Preparation of Magnetic Filler Particles

**Figure 2:**
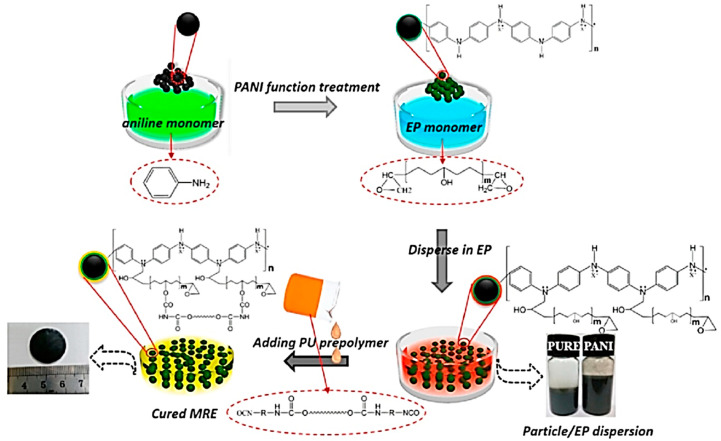
Schematic of surface modification mechanism of carbonyl iron particles (CIPs) with Polyaniline (PANI) coating and fabrication of PANI-modified CIP-based isotropic MREs [91] (reprinted with permission from Elsevier^TM^).

Sometimes, magnetic filler particles are prepared for improving surface properties or altering the shape of particles (sphere, flower, or plate) to improve the interfacial and MR properties of MREs [78,91,96]. The surface or interfacial properties of magnetic filler particles (CIPs) can be improved by depositing Polyaniline (PANI) coating on their surfaces. PANI coating modifies the surface of CIPs through in situ chemical oxidative polymerization process, using ammonium peroxodisulfate (APS) as an oxidant and p-toluenesulfonic acid (p-TSA, C_7_H_8_O_3_S) as a dopant. The schematic of the surface modification mechanism of CIPs with PANI coating and fabrication of PANI-modified CIP-based isotropic MREs are illustrated in Figure 2 [91]. Table 1 shows various types of elastomeric matrices, magnetic filler particles, additives, and key parameters—used in the fabrication of isotropic MREs.

**Figure 3:**
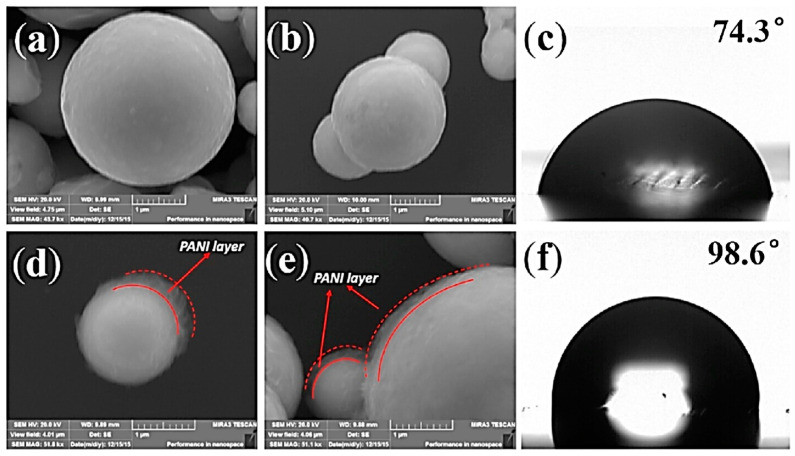
SEM micrographs of (**a**,**b**) pure CIPs and (**d**,**e**) PANI-modified CIPs; contact angles of a water droplet on (**c**) pure CIPs and (**f**) PANI-modified CIPs [91] (reprinted with permission from Elsevier^TM^).

The surface modification mechanism of CIPs by PANI-coating involves the preparation of a solution of 1.8 mmol p-TSA in 240 mL of distilled water and mixing of specific amounts of CIPs and Aniline (Ani) in this solution with ultrasonication for 30 min under the ice–water bath. A 36-mmol APS aqueous solution is then mixed in this solution and left with vigorous stirring under an ice–water bath for 14 h to promote polymerization. PANI-modified CIPs are collected after washing with ethanol and distilled water several times and drying at 60 °C for 24 h under vacuum. SEM micrographs of pure and PANI-modified CIPs are illustrated in Figure 3. These micrographs demonstrate the spherical shape of both pure and PANI-modified CIPs. They also confirm successful preparation of the gauzelike PANI coating of thickness 50–200 nm on CIPs [91].

On the other hand, the shapes of filler particles (CIPs) can be altered from sphere to plate by a specific milling process. In this process, a rotary type ball mill and zircon balls as a grinding media are used in the ratio of 20:1. Additionally, pure ethanol is added as a control agent to prevent particle adhesion and to improve process efficiency. Sphere-shaped CIPs are milled in this rotary mill for 40 h and operated at a speed of 380 rpm to transform their sphere shape into plate shape [78].

### 2.2. Fabrication of Isotropic Magnetorheological Elastomer

In the case of SR, the fabrication of isotropic MREs is quite easy and simple. The first step in SR-based MRE fabrication is the thorough mixing of liquid SR with suitable additives, such as catalyst [73], silicone oil (SO) [31], PDMS [45], graphene nanopowder [97], or 1,3-divinyl-1,1,3-tetramethyl disiloxane [22], and suitable magnetic filler particles for sufficient time to obtain a uniform dispersion of particles in the solution [45]. The magnetic filler particles are usually added in the volume fractions, ranging from 5–40 vol%, whereas additives are added as per the required properties. Afterwards, this blend is placed inside a vacuum chamber for sufficient time to remove the trapped air bubbles during mixing [73]. After proper degassing, the blend is poured into plastic or metal molds. Then, the molds are again placed in the vacuum chamber for sufficient time for further degassing. As mentioned earlier, SR has the property of room temperature vulcanization, therefore, the solution is cured (vulcanized) in the molds at room temperature [23] or slightly higher temperature (65 °C) [95] after sufficient time, ranging from 10–2880 min [35,45]. In the case of any other polymeric matrix, high-temperature vulcanization is carried out.

On the other hand, the PUR-based MREs are fabricated by mixing PUR rubber with additives (SO) and magnetic filler particles (CIPs) thoroughly at room temperature [12] or a slightly higher temperature (67 °C) [61]. This mixture is then poured into molds and cured at a specific temperature and pressure (20 KNm^−2^) to get a final product [12,61]. Similarly, NR-based MREs are fabricated by mixing NR with filler particles homogeneously by two-roll mill or any other means and pouring into molds. Curing of NR-based MREs is performed in an oven, maintained at 180 °C under specific pressure (200 bar) for a specific time (10 min) to get the final isotropic MRE [27]. Furthermore, the fabrication of isotropic MRE from scrap tire rubber involves the separation of rubber from metals and fabrics, shredding into powder of 60-mesh size, and analyzing its chemical composition (usually 7% acetone extract, 5.45 ash, 32.9% carbon black, 54.6% hydrocarbon rubber). The Fe_3_O_4_ (60-mesh size) or Penta CIPs (6 μm) particles in 10–40 wt% are usually used as magnetic filler particles and sulfur, zinc oxide, stearic acid, and latex solutions as additives. The fabrication process of MREs from scrap tire rubber is comprised of several stages, including mixing of 100 phr of crumb rubber with 2 phr of sulfur, 1.5 phr of stearic acid, and 5 phr of zinc oxide for 15–30 min; addition of 15% latex solution; and further mixing for 15 min. Finally, the mixture is poured into molds and placed in a high-pressure high-temperature (HPHT) sintering device [92]. Sintering of molds was performed by applying a pressure of 25 MPa, heating to a temperature of 200 °C at a heating rate of 10 °Cmin^−1^ within 17–20 min, and soaking at this temperature for 1 h. The hot molds are then cooled to room temperature. The volume fraction of produced MREs can be derived using Equation (1) [33].

(1) $$\varphi \;=\;\frac{d_{MRE}\;-\;d_{W}}{d_{MP}\;-\;d_{W}},$$

where *d_MRE_* is the density of MREs, *d_W_* is the density of pure reclaimed rubber, and *d_MP_* is the density of magnetite powder. The base densities for the pure reclaimed rubber and magnetite powder were 1.107 gcm^−3^ and 5.27 gcm^−3^, respectively [33,92].

## 3. Properties of Isotropic Magnetorheological Elastomers

### 3.1. Morphological Properties

**Figure 4:**
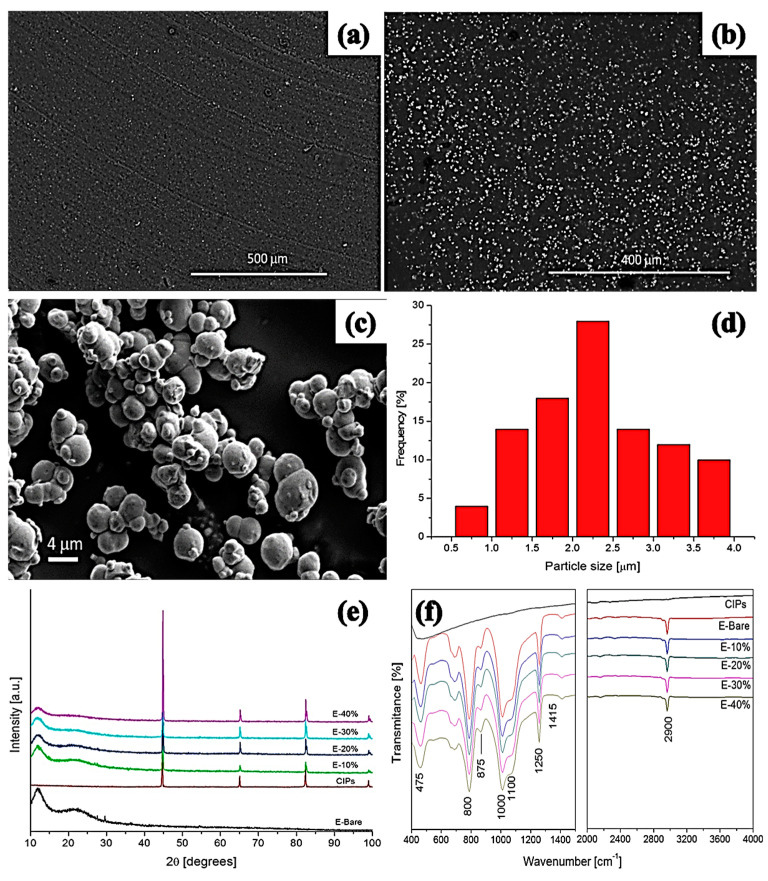
SEM micrographs of (**a**) CIPs-free MRE, (**b**) MRE with 20 wt% CIPs, (**c**) high-magnification micrograph of MRE, (**d**) histogram exhibiting CIPs size distribution in MREs, (**e**) XRD spectra, and (**f**) FTIR spectra of MREs [23] (reprinted with permission from Elsevier^TM^).

**Figure 5:**
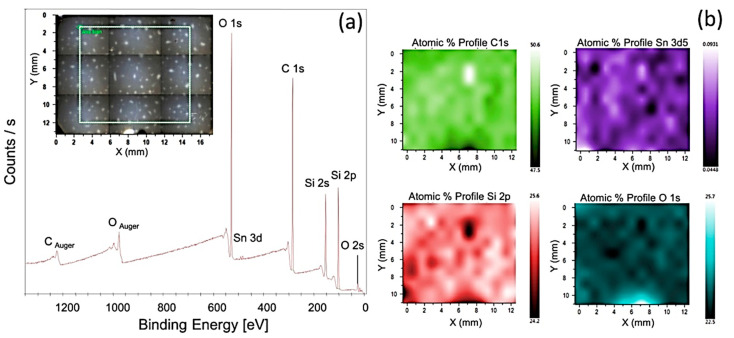
(**a**) XPS spectra of MRE with 20 wt% CIPs, demonstrating carbon, oxygen, silicon, and tin elements in the selected area, presented in inset figure. (**b**) XPS maps and atomic percentages of elements for the selected area [23] (reprinted with permission from Elsevier^TM^).

Various characterization techniques—i.e., scanning electron microscopy (SEM), energy dispersive spectroscopy (EDS), X-ray photoelectron spectroscopy (XPS), Fourier transform infrared spectroscopy, and X-ray diffraction spectroscopy (XRD)—have been employed to explore the morphological properties of isotropic MREs. It has been reported that the morphology of SR- and CIP-based MREs comprises homogeneously distributed CIPs throughout the SR matrix [34]. The large size CIPs of 6.25-µm diameter exhibit fairly uniform distribution, whereas small diameter CIPs produce agglomerates in the matrix. In the case of small diameter CIPs, the distance between particles was observed to be smaller and filler–filler particle interactions was greater, resulting in agglomeration of particles [99]. The addition of 10 wt% silicone oil causes more homogeneous dispersion of CIPs in the SR matrix without any structuring or surface defect [29]. SEM images, a histogram of particle size, XRD, FTIR spectra of SR, and CIP-based MREs, having PDMS as an additive, are illustrated in Figure 4. It was found that the morphology of CIPs-free MRE validated the formation of nonporous composite elastomers due to vulcanization under vacuum conditions (Figure 4a). On the other hand, Figure 4b demonstrated the random distribution of CIPs in the SR matrix. Similarly, Figure 4c,d confirmed the random distribution of CIPs with 2.5-mm average particle diameter in the SR matrix in the range of 0.5–4 mm. Two broad peaks at 12° and 23° in the XRD spectra validated the presence of PDMS and amorphous nature of SR-based polymer composite, whereas intense peaks at 44.8°, 65°, and 82.3° confirmed the presence and crystalline nature of CIPs (Figure 4e). The FTIR spectra of this MRE confirmed the asymmetric stretching vibration motion of Si–O–Si bond by absorption band at 800 cm^−1^ and the bending motion of Si–OH group by a peak near to 875 cm^−1^ (Figure 4f). The stretching vibrations of Si–O, and Si(CH_3_)_2_ groups were also validated by peaks in the range of 1000−1100 cm^−1^. The additional absorption bands in the region of 1250 cm^−1^ and 2960 cm^−1^ presented the stretching vibration of Si(CH_3_)_2_ [23]. XPS spectra verified the results of FTIR spectroscopy, as illustrated in Figure 5.

It has also been reported that the CIPs of 4–5 μm diameter exhibit uniform distribution in EPDM- and CIP-based MRE, whereas CIPs of diameter 8−10 μm demonstrate aggregation of 2–3 particles. This aggregation begins with the addition of 10 phr of CIPs and 60 phr of carbon black in the EPDM. The MRE with 5 phr of CIPs demonstrated the fair distribution of single CIP within the EPDM matrix without aggregation. In two separate MREs, containing 30 phr of CIPs and 30 phr of BIPs of a diameter of 4–5 μm, a combination of single-particle distribution and aggregation of 2–3 particles was observed. But the MRE containing 30 phr of CIPs demonstrated a very homogeneous distribution compared to BIP-based MRE [24]. Cvek et al. [30] reported the uniform dispersion of CIPs in both virgin and reprocessed thermoplastic elastomer (TPE)-based matrix CIPs without agglomeration and air bubbles. On the other hand, the morphology of PUR-based MREs, containing pure CIPs and PANI-modified CIPs, have also been explored. Compared to pure CIP-based MREs, homogeneous and agglomerated free morphology was achieved in the PANI-modified CIP-based MREs. A particular self-assembled structure of modified CIPs and excellent compatibility between the CIPs and PUR was observed in the PANI-modified CIP-based MRE, attributed to the bridging of the CIPs/PUR covalent bonds [91].

### 3.2. Particle Distribution

**Figure 6:**
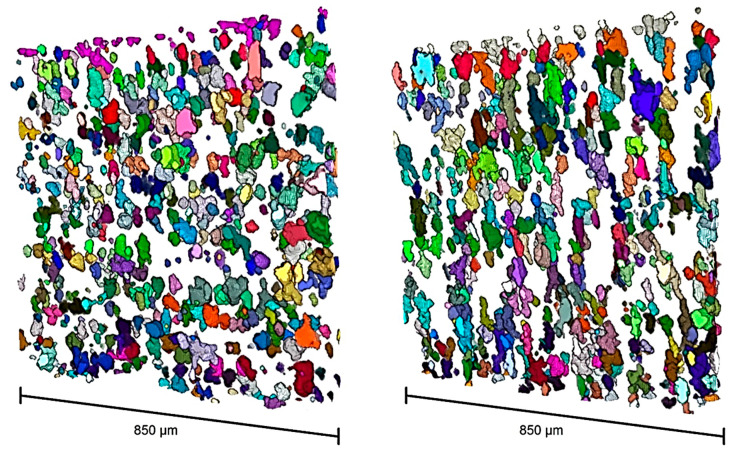
The 3D reconstructed images of tomography data after particle separation. (**Left**) Initial microstructure of particles distribution under 0-mT magnetic field and (**Right**) microstructure of the same sample under 250-mT magnetic field with particles chains [22] (reprinted with permission from Elsevier^TM^).

Arrangement of particles within the matrix has been reported to have a great influence on the macroscopic properties of MREs and is considered a key parameter in tailoring MREs [100,101]. X-ray computed microtomography (CT) has been proved as a reliable and accurate method of analyzing the arrangement of the particles in the matrix of MREs [102,103]. The distribution of CIPs in the SR matrix has been evaluated using the CT technique. No clear alteration of the CIPs distribution in the SR matrix was observed after the application of a 10-mT magnetic field or 12% strain. But the application of a 10-mT magnetic field caused significant particle rotation and increased the number of small-angle particles towards the magnetization axis. Whereas, the application of strain forced back the particles to their initial state to some extent and reduced the amount of small-angle particles. At 250-mT magnetic field, the CIPs aligned into the chainlike structure and the number of small-angle particles increased, as illustrated in Figure 6 [22].

On the other hand, elongated and oblate-shaped particles were observed in the microstructure of PDMS- and CIP-based MREs, which were challenging for sieving and resulted in an overlap of the size fractions. The sphere-shaped particles were observed by laser diffraction measurements, whereas the largest ellipsoid dimensions of the particles were observed by CT, but overall identical particle size distribution was observed through both techniques. A classification of the shape of particles was conducted, similar to the work of Zingg et al. [104,105]. A broad distribution of aspect ratios was also observed without preferred oblate and prolate shapes. The classes of small size particles feature less extreme aspect ratios, preferring spherical shapes [77]. It has been reported that the dispersion of magnetic filler particles as well as resulted properties of isotropic MREs strongly depend on the size, shape, and volume fraction of magnetic filler particles. Due to the bonding nature and agglomeration of magnetic filler particles, small-sized particles exhibit a better reinforcing effect than large-sized particles. Agglomeration of small-sized particles occurs due to greater filler–filler particle interaction and lower matrix–filler interaction [31]. Similarly, at a lower volume fraction of magnetic filler particles, the distance between the particles remains larger, and particles disperse homogeneously throughout the matrix without agglomeration. With the increase of volume fractions of particles, the distance between the particles decreases and results in poor dispersion, greater agglomeration, and poor properties. This might also be attributed to the greater filler–filler particle interaction and lower filler–matrix interaction at a higher volume fraction of magnetic filler particles [99]. In the fabrication of isotropic MREs, homogenous dispersion of filler particles is usually achieved by the use of appropriate additives, such as slackers or silicon thinner, or by ultrasonication for sufficient time. The distance between the particles has a significant impact on particle dispersion in the polymer matrix. The interparticle distance (H) can be calculated by Equation (2) [106]:

(2) $$\frac{H}{d}\;=\;{\left[\frac{\varphi_{max}}{\varphi }\right]}^{\frac{1}{3}}\;-1,$$

where “*d*” is the diameter of particle, “φ” is the volume fraction of the particle, and “$\varphi_{max}$” is the volume fraction at maximum random packing (≡0.64) [106].

### 3.3. Mechanical Properties

The mechanical properties, including Shore A hardness, tensile strength, elongation, and modulus, without the application of the magnetic field, can be evaluated by shore A hardness and tensile tests. Tensile testing of SR- and CIP-based MREs showed that the addition of 10%, 20%, and 30% of CIPs caused 34%, 95%, and 58% improvement in tensile strength, compared to bare SR of Shore A hardness 25. Although the addition of 20% CIPs caused maximum improvement in tensile strength, it also caused a 24% reduction in tensile modulus. Contrary to the work of Farshad and Benine [107], an unexpected reduction in the stiffness of MREs was also observed after the addition of CIPs in SR. This might be associated with the use of CIPs of smaller diameter and SR of low hardness. Further, according to the Flory statistical theory of rubber elasticity, the addition of CIPs decreases the mass fractions with cross-linked structures, reduces the number of cross-links, and thus reduces the stiffness of MREs [23].

**Figure 7:**
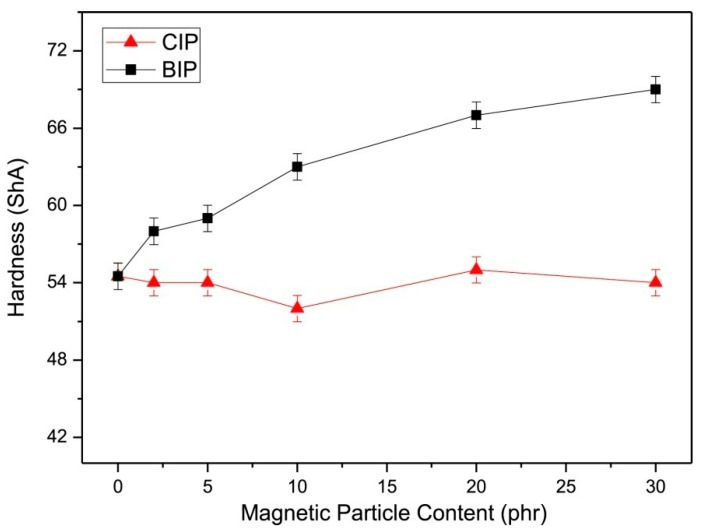
The plot of shore A hardness as a function of magnetic particle content (phr) of EPDM-based MREs containing BIPs and CIPs [24] (reprinted with permission from Elsevier^TM^).

Similarly, the mechanical properties of EPDM-based MREs with varying content (phr) of BIPs and CIPs were also evaluated. The variations in their shore A hardness values with varying content (phr) of BIPs and CIPs are plotted in Figure 7. It was observed that the hardness of the EPDM-based MREs increased with increasing BIPs content, whereas increasing content of CIPs caused no considerable change in hardness. Conversely, the EPDM-based MRE with 5 phr of CIPs exhibited significantly higher tensile strength and elongation than the MRE with 5 phr of BIPs. Increasing content of CIPs and BIPs between 10–30 phr decreased the tensile strength and elongation but increased the elastic modulus, which can be attributed to the particle agglomerations. An optimum combination of mechanical properties was exhibited by MRE with 5 phr of CIPs. MRE-incorporated BIPs exhibited much higher elastic modulus than MREs incorporating CIPs [24].

**Figure 8:**
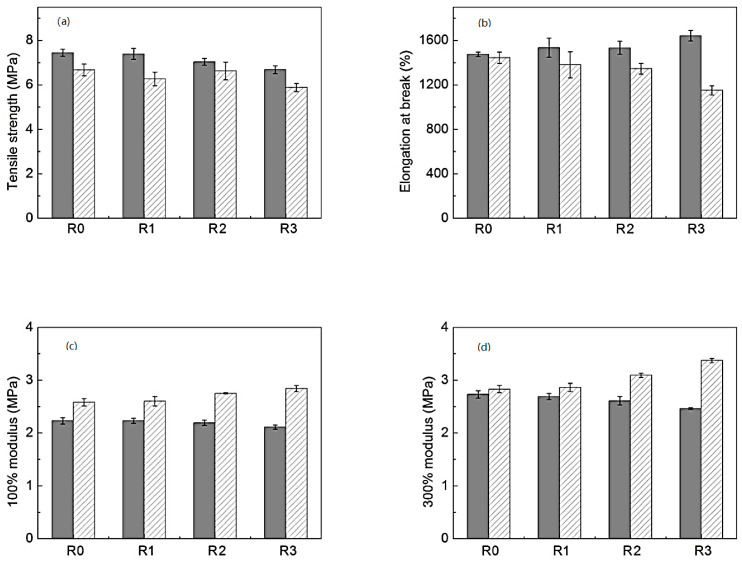
Properties of the pure thermoplastic elastomers (TPEs) (dark) and TPE-based MREs (stripped) subjected to various processing cycles (R0–R3) (**a**) Tensile strength, (**b**) Elongation at break, (**c**) 100% Modulus, (**d**) 300% Modulus [30] (reprinted with permission from Elsevier^TM^).

On the other hand, the tensile properties of pure TPEs and TPE-based MREs, subjected to several processing iterations, are plotted in Figure 8. It was observed that the tensile strength of pure TPE decreased by ~10% and elongation at the break increased by ~11% after the final last processing iteration. This might be due to a decrease in *M*_n_ and the development of scarce transversal cross-links after processing, respectively [108]. Conversely, the tensile strength of TPE-based MREs remained virtually unaltered, but a decrease of ~20% in elongation at the break was observed after the final processing iteration. This unexpected behavior might be attributed to the interfacial particle/matrix bonding [108]. As per processing-induced particle/matrix bonding theory, TPE-based MREs demonstrated slightly better modulus than pure TPEs, particularly after the last processing iteration, their modulus enhanced by ~11−19% [30].

### 3.4. Dynamic Mechanical Properties

**Figure 9:**
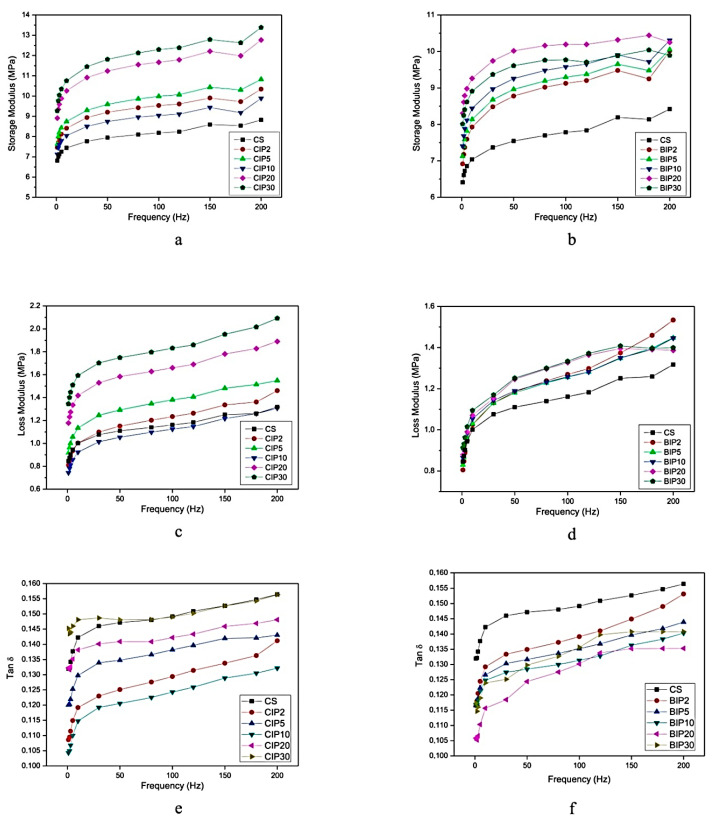
Dynamic properties of EPDM-based isotropic MREs, including storage modulus. (**a**) MRE containing CIPs; (**b**) MRE containing BIPs, loss modulus; (**c**) MRE containing CIPs; (**d**) MRE containing BIPs, and Tan δ; (**e**) MRE containing CIPs; (**f**) MRE containing BIPs as a function of frequency [24] (reprinted with permission from Elsevier^TM^).

The dynamic mechanical analyzer is usually used to evaluate the dynamic mechanical properties of MREs, including storage modulus, loss modulus, and damping factor (tan δ), as a function of frequency and amplitude. The dynamic mechanical properties of EPDM-based MREs with CIPs and BIPs were evaluated without the application of the magnetic field, their frequency dependence is illustrated in Figure 9. It was found that the storage modulus values of both MREs increased with the increase of frequency as well as CIPs and BIPs contents. But the MRE with CIPs demonstrated comparatively higher storage modulus than MREs with BIPs (Figure 9a,b). The addition of 2 phr and 5 phr of CIPs produced greater storage modulus than 10 phr of CIPs. But further addition of 20 phr and 30 phr of CIPs significantly increased the storage modulus for all frequencies. This can be attributed to the load transfer between CIPs and EPDM matrix. Conversely, the storage modulus of MREs with BIPs increased systematically with BIPs addition. The loss modulus values of both MREs, as a function of frequency, are plotted in Figure 9c,d, respectively. The loss modulus increased with an increase in frequency, with a significantly higher slope compared to the storage modulus. The MRE with 10 phr of CIPs offered lower loss modulus than all the samples, even lower than the control sample, for all frequencies. However, a further increase in CIPs content to 20 and 30 phr caused a drastic increase in loss modulus. The tan δ of both the MREs was observed to increase with increasing CIPs and BIPs content, as illustrated in Figure 9e,f. It is important to note that MREs with 2−10 phr of BIPs demonstrated a considerably greater value of tan δ than MREs with CIPs, for all frequencies. On the other hand, increasing strain amplitude up to 2.5% caused such a decrease in storage modulus and increase in tan δ that they reached a plateau region, which might be attributed to the loosening of CIPs and BIPs within the EPDM matrix [109]. It was observed that the addition of 2−10 phr of both CIPs and BIPs resulted in much lower storage modulus values than the control sample, whereas 20–30 phr addition caused much higher values, for all strain amplitudes [24].

### 3.5. Thermal Properties

The evaluation of the thermal properties of MREs is also very important to estimate the effect of atmospheric temperature on MREs. Therefore, the thermal degradation of waste tire rubber and Fe_3_O_4_-particle-based MREs were evaluated by differential scanning calorimetry (DSC). The DSC curves of MRE samples contained varying Fe_3_O_4_ content and pure reclaimed scrap tire rubber. The transition from the glassy phase to the rubbery phase through endothermic reaction occurred between −65 °C to −55 °C. The measured glass transition temperatures (*T*_g_) of all MREs were −0.6 °C ± 0.5 °C [110]. Although the Fe_3_O_4_ content demonstrated no significant effect on *T*_g_ values, it considerably improved the thermal conductivity of MREs compared with pure reclaimed tire rubber. Consequently, increasing the fraction of Fe_3_O_4_ particles increased the thermal conductivity, as presented by upshift in the DSC curves for all samples [33].

### 3.6. Magneto-Mechanical Properties

**Figure 10:**
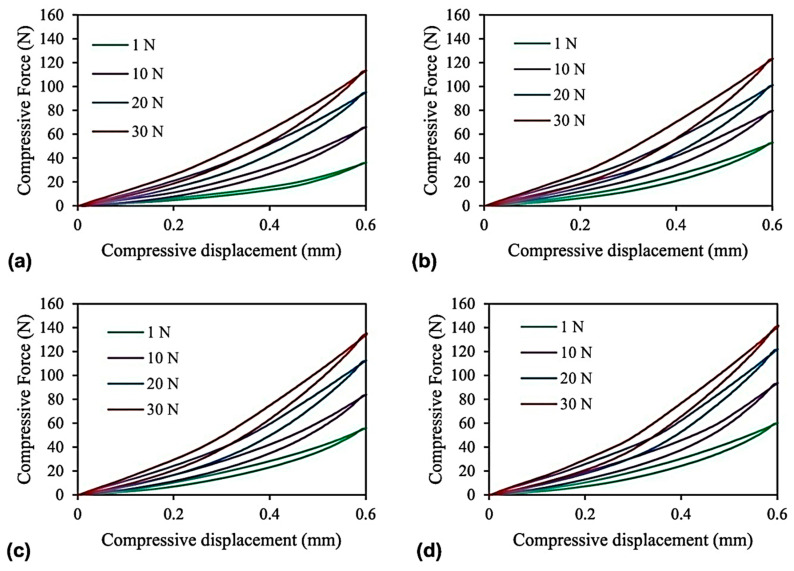
The plot of compressive force–cyclic compressive displacement under the conditions of constant peak-to-peak displacement (0.6 mm), constant frequency (0.1 Hz), various preloads, and various magnetic field strengths: (**a**) 0 mT, (**b**) 190 mT, (**c**) 320 mT, (**d**) 520 mT [89] (reprinted with permission from Elsevier^TM^).

Magneto-mechanical properties of MREs present the deformation behavior of MREs in the presence of a magnetic field under tensile or compressive forces. Magneto-mechanical properties of SR- and CIP-based MREs, under the conditions of the constant peak-to-peak displacement (0.6 mm), constant frequency (0.1 Hz), and various preload and magnetic field strengths, are illustrated in Figure 10. It was observed that increasing preloading separated the force–displacement curves, made the curves steeper, increased the area enclosed by the hysteresis loops, and thus increased the damping capability and stiffness of MREs. Moreover, the relative shift in the curves of preload without the magnetic field looked comparatively greater than with the magnetic field, presenting a strong impact of preload without the magnetic field [89].

**Figure 11:**
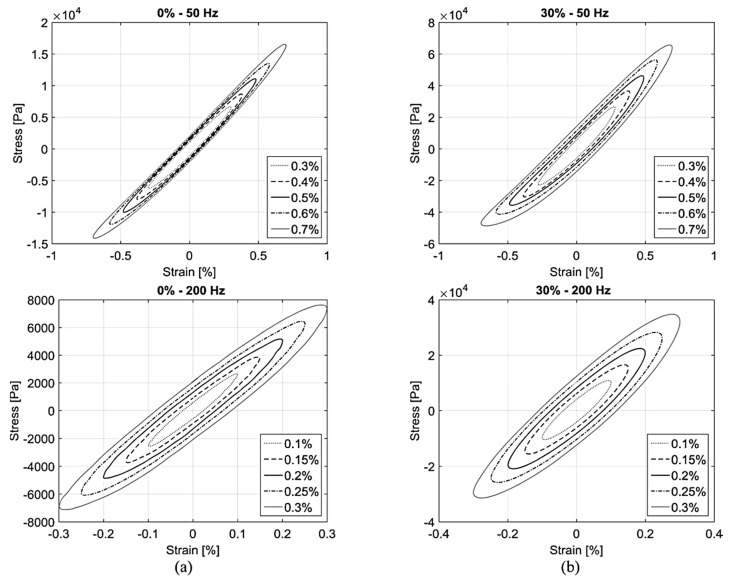
Stress–strain diagrams of MREs with CIPs concentration (**a**) 0% and (**b**) 30% under frequencies of 50 Hz and 200 Hz [27] (reprinted with permission from Elsevier^TM^).

The stress–strain diagrams of NR- and CIP-based MREs are illustrated in Figure 11. It can be observed that within the linear viscoelastic (LVE) region, the increasing strain made the amplitude of ellipses wider and larger without altering the shape. Under lower frequency and higher CIPs concentration conditions, the elliptical shape disappeared with strain amplitude. Furthermore, increasing CIPs concentrations from 0% to 30% caused a 75% improvement in the storage modulus by 0% filler reinforcement mechanism [111], similar to the work of [109]. Conversely, MRE with 15% and 30% CIPs demonstrated a decrease in loss factor. This can be attributed to the development of internal particle friction and interfacial damping between the particles and matrix by the introduction of CIPs to the polymeric matrix, which improves the damping of MRE produced by polymeric chains [112,113,114]. The evaluation of the effect of magnetic field strength on storage modulus of an MRE with 30% CIPs showed that increasing the magnetic field strength increased the storage modulus. The MRE with a higher CIPs concentration exhibited a larger MR effect under higher magnetic field strength but not as large as reported in the literature in compression mode [115,116,117] due to lower magnetic field strengths. In addition, the MR effect in the compression mode was observed to be greater than in the shear mode [27].

On the other hand, the magneto-mechanical properties of PDMS- and fractionized IP-based MREs were also evaluated and analyzed under various compressions. It was found that the first compression resulted in an irreversible deformation, whereas the subsequent compression as well as compression without the magnetic field offered no irreversible deformation. Increasing the size of IPs considerably decreased the moduli without the magnetic field, whereas it maintained the moduli constant with the magnetic field. The decrease in moduli may be attributed to the particle/matrix interaction [77]. For a constant mass fraction of IPs, increasing particle size decreases particle surface and increases the MR effect. Small IPs having a large surface increased the overall stiffness and Young’s modulus of the MRE [118,119]. The Young’s modulus and corresponding MR effect of the extremely soft SR matrix and magnetically soft CIP-based MRE were determined. This MRE offered an extraordinary large MR effect (633% ± 55%), which can be associated with the high concentration of CIPs and extremely low hardness of matrix. This MRE also demonstrated the highly reversible Young’s modulus, which returned to its initial value right after the removal of the magnetic field. It was concluded that the MRE possessed high elasticity and low viscosity [22].

#### Magneto-Shear Properties

Magneto-shear properties describe the deformation behavior of MREs in the presence of a magnetic field under shear stress. Magneto-shear properties of SR- and CIP-based MREs were evaluated under both static and dynamic shear loads. The results of static tests performed on all six types of MREs under various magnetic field strengths (0–450 mT) showed that stiffness of the MREs increased with increasing magnetic field strength, as previously reported [120,121]. MREs exhibited nearly linear stress–strain curves in the absence of a magnetic field, whereas nonlinear curves in the presence of a magnetic field were more prominent at 540 mT. The increasing volume fraction of CIPs also contributed to improving nonlinearity behavior. Increasing strains decreased the slopes of stress–strain curves, particularly at higher magnetic fields (450 mT), attributed to the strain-softening properties of filled polymeric matrix [122]. This effect is more prominent at a higher volume fraction of CIPs. The strain-stiffening effect associated with saturation of the chains of polymers or the limited extensibility plays a significant role in improving the shear modulus of MREs, as previously reported [123,124,125]. Although the static shear modulus of all samples of MREs improved with the increase of the strength of the magnetic field, the MRE with the highest CIPs concentration (40%) exhibited the highest value among all. The results also revealed the saturation of static shear modulus of this sample exceeding 300 mT, similar to the works of [95,126,127]. 

**Figure 12:**
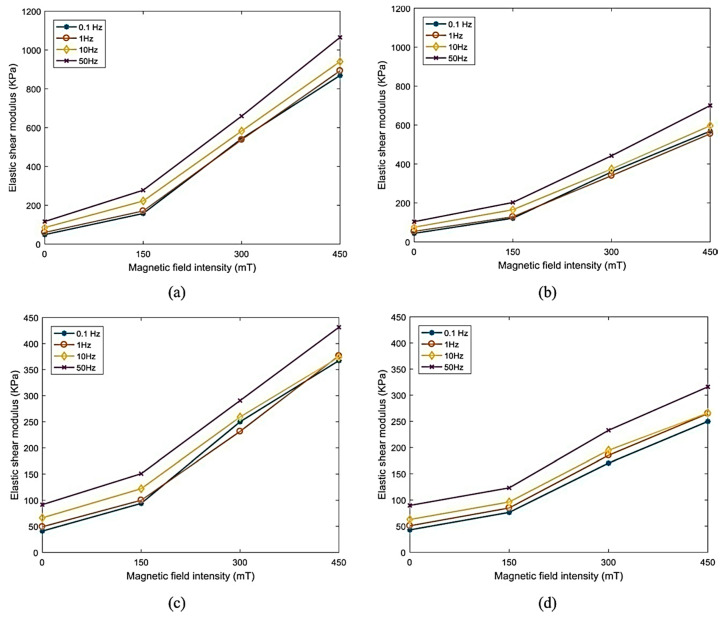
Impact of magnetic flux density on elastic shear modulus under various strain amplitudes and various excitation frequencies: (**a**) 2.5%, (**b**) 5%, (**c**) 10%, (**d**) 20% [95] (reprinted with permission from Elsevier^TM^).

On the other hand, the dynamic magneto-shear properties of MRE having 40% SR, 40% CIPs, and 20% additives were also evaluated. Perfectly elliptical hysteresis loops and the almost viscoelastic response were observed without a magnetic field. An increase in slope and nonelliptical hysteresis loops of stress–strain curves with increasing frequency under a magnetic field ranging from 0−150 mT presented considerable sensitivity of damping and effective stiffness of the MREs. The nonlinearity becomes more significant under a higher magnetic field strength (300 and 450 mT) associated with the strain-softening effect. Higher magnetic field strength also caused a greater increase in loss modulus than the storage modulus of MRE irrespective of the strain amplitude. Improvement in strain amplitude exponentially decreased the storage modulus and loss modulus without the magnetic field, whereas they decreased significantly with the magnetic field irrespective of the excitation frequency and magnetic field strength. Under higher strain amplitude, the MRE behaved like a nonlinear viscoelastic material. It was also observed that the elastic shear modulus decreased with increasing excitation amplitude and significantly decreased under higher magnetic field strength. The impact of varying magnetic flux density on elastic shear modulus under ranges of excitation frequency and strain amplitude of the MRE having 40% SR, 40% CIPs, and 20% additives is illustrated in Figure 12. It was observed that irrespective of the strain amplitude and excitation frequency, elastic shear modulus increased considerably with the increase in magnetic flux density. Under lower values of excitation frequency and strain amplitudes, elastic shear modulus increased significantly. Under 2.5% strain amplitude and 0.1 Hz frequency up to 1672%, increased storage modulus was observed. This tremendous variation in the relative MR effect of this MRE having a higher concentration of CIPs presented that this MRE can be used in applications of controllable vibration absorbers and isolators [95].

Magneto-shear properties of SR- and CIPs-based MREs were investigated by lap-shear instrument (Experiment). Under smaller shear deformation less than 0.25, an almost linear relationship was observed between shear stress and shear strain. Increasing magnetic field strength increased the slope of shear stress–strain curves whereas increasing concentration of SO slightly decreased it. The higher concentration of SO in MRE caused more softening effect in the MRE matrix [29,128].

Stress–strain relationships of MREs having 7.5 wt% SO under gradient magnetic field were also evaluated. Approximate linear stress–strain curves were obtained, and the slope of these curves increased with an increase in the magnetic field similar to curves obtained under a uniform magnetic field. Similar results were obtained for two permanent magnets of different strengths (PM100 and PM50). Moreover, an almost linear slight increase in shear stress was observed with increasing strength of the magnetic field at higher strain under smaller deformation. The load-bearing ability of this MRE was higher in the presence of PM100 (lower gradient magnetic field) than PM50 (higher gradient magnetic field) under the same shear strain and magnetic field. Shear modulus also showed higher sensitivity to magnetic field strength, SO concentration, and gradient of the magnetic field. Shear modulus considerably improved with increasing magnetic field strength and decreased with the increase in SO concentration. PM100 caused considerable strengthening of modulus of magnetic induction, compared to PM50. This is associated with the mean magnetic field strength within the sample reduced under a magnetic field of high gradient, resulting in a reduction of the magnetic energy density input. The MR effect also exhibited sensitivity to both SO concentration and gradient of the magnetic field. The MR effect of MREs increased with an increase in SO concentration but decreased with an increase in magnetic field gradient, attributed to the lower mean magnetic field intensity under higher magnetic field gradient. Considerably larger magneto-induced properties can be achieved in MREs by the addition of higher fractions of magnetic particles and by selecting a matrix with lower elastic modulus [29]. 

### 3.7. Rheological Properties

Rheological properties of bare and 20% CIP-reinforced SR- and PDMS-based MREs were investigated through parallel plates configuration at a controlled angular strain of 1% and a frequency of 1 Hz. The addition of CIPs in MREs caused a considerable decrease in storage modulus from 19.5 to 9.6 kPa and tan δ from 39.8% to a strain of 25% presenting fluidlike behavior with less strain of CIPs compared to the bare polymer. Their addition also reduced the complex viscosity and viscosity related to dynamic rigidity and diminished the MRE capacity of storing energy. Variations in frequency also significantly alter the rheological properties of MREs as the highest value of tan δ was achieved at 63.1 Hz for bare polymer, whereas for 20% CIP-reinforced MRE, the highest value of tan δ was achieved at 19.95 Hz. These MREs behaved like solids as the tests were performed at a strain of 1% and the achieved values of tan δ were not close to 1. The effect of change in intensity of magnetic field on rheological properties of MREs was also evaluated by varying between 7 mT to 1000 mT. It was observed that increasing magnetic field intensity increases the storage modulus. Even at a very-small magnetic field intensity, a considerable increase can be observed in storage modulus from approximately 9 kPa to 53.7 kPa. The addition of more CIPs to the SR matrix caused improvement in MREs deformational energy and reduction in shear modulus compared to bare polymer [23].

**Figure 13:**
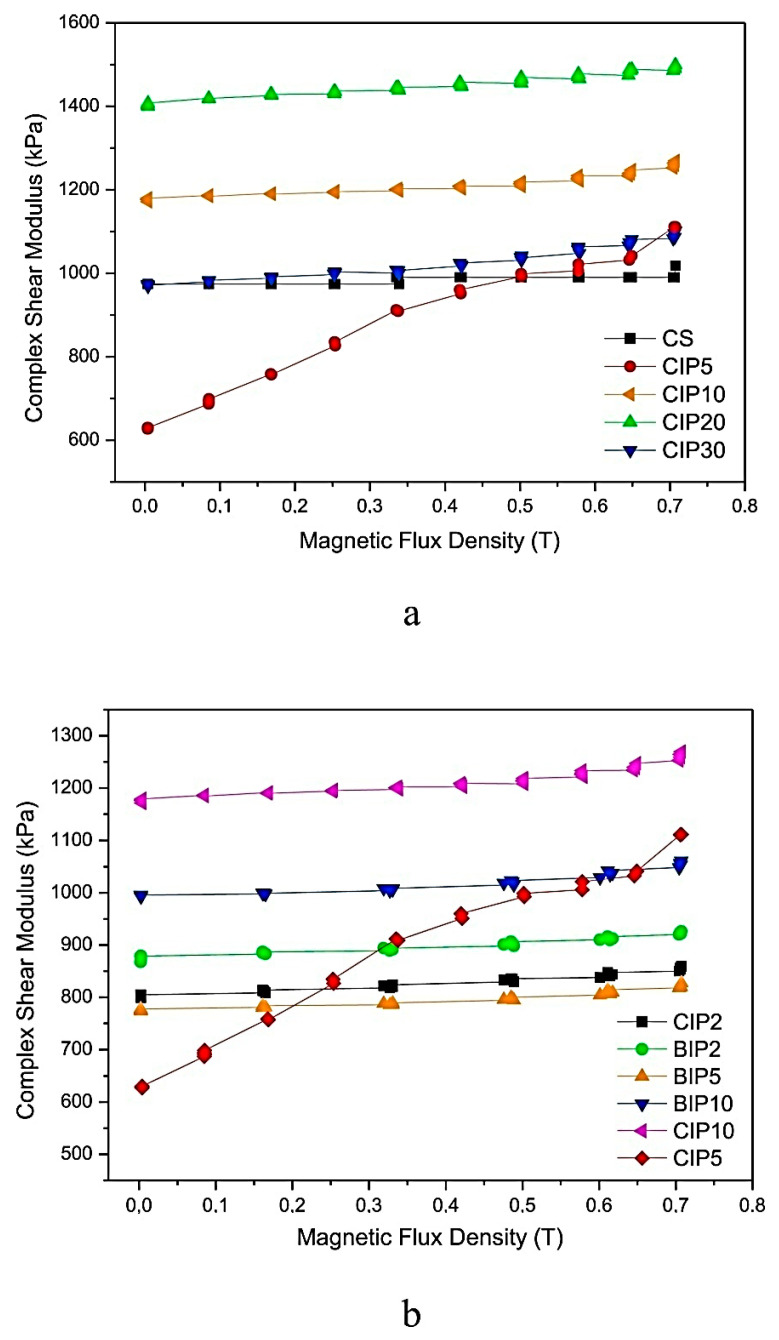
The plot of complex shear modulus vs. magnetic flux density of EPDM-based MREs with warious concentration of CIPs and BIPs (**a**) 5–30 phr of CIPs, (**b**) 2–10 phr of CIPs and 2–10 phr of BIPs [24] (reprinted with permission from Elsevier^TM^).

EPDM-based MREs containing various percentages of CIPs were observed to be highly sensitive to the magnetic field. Their complex shear moduli was measured under varying magnetic flux density and plotted in Figure 13. Increasing magnetic flux density caused no change in the complex shear stress of the control sample having zero percentage of CIPs but increased the complex shear modulus of MREs samples having various percentages of CIPs. Among all, MRE containing 5 phr of CIPs exhibited the highest sensitivity to magnetic flux density and the highest improvement in complex shear modulus with an increase in magnetic flux density. This is due to the fact that the highest EPDM/CIPs interaction was present in this MRE (with 5 phr of CIPs). Under the application of a magnetic field, CIPs push each other and well-oriented CIPs develop stress in the cross-linked vulcanizate, thus increasing the complex shear modulus. On the other hand, MREs having 2−10 phr BIPs demonstrated no improvement in complex shear modulus under varying magnetic fields. MREs with 5 phr of CIPs also showed the highest (77%) MR effect among all samples, whereas MREs with 2−10 phr BIPs did not demonstrate a considerable improvement in MR effect. The major reasons behind the lower complex shear modulus and lower MR effect of MREs with BIPs are their higher elastic modulus, higher hardness, higher Pyane effect, higher tan δ values, and lower elasticity value compared to MREs with CIPs [24].

The storage modulus values of reprocessed TPE-based MREs were firstly analyzed under various strains. At lower strains, the storage modulus of these MREs exhibited strain independency defining the linear viscoelasticity region (LVR), whereas at high strains, it showed a decrease in storage modulus presenting permanent deformation of MREs sample. Each reprocessing cycle caused a significant increase in the storage modulus of these MREs. In the LVR region, both the reprocessing cycles and applied magnetic field had zero effect [30].

On the other hand, it has been reported that elasticity of matrix considerably affects the restructuration of particles and thus, the MR effect given by the Equation (3) [129]:

(3) $$\mathrm{Relative}\;\mathrm{MR}\;\mathrm{Effect}\;=\;\frac{\left(G_{H}^{\prime }\;-\;G_{0}^{\prime }\right)}{G_{0}^{\prime }},$$

where $G_{H}^{\prime }$ and $G_{0}^{\prime }$ are the magnetic field on and magnetic field off storage moduli, respectively. The relative MR effects were calculated at 1-Hz frequency and 288-kAm^−1^ magnetic field strength providing the values of 68%, 46%, and 29% for the reprocessed samples, respectively. These values are the result of lower relative CIPs motion within the TPE-based reprocessed MREs [130]. Reprocessing was observed to decrease the MR effect of TPE-based MREs. Reprocessing of MREs could be utilized to preserve significant mechanical and rheological properties after a large number of cycles [30].

Both the storage and loss moduli of pure and PANI-modified MREs measured under various frequencies ranging from 0.1−100 Hz at fixed 0.1% amplitude have also been investigated. It can be seen that both the storage modulus and loss modulus of PANI-modified MREs are greater than pure MREs. Due to mismatching between the relatively low-speed movement of polymeric chains and the fast shear force applied to the sheet matrix, the storage modulus of all MREs improved with the increase in frequency with and without the magnetic field [131]. Increasing frequency also decreased the dynamic response time of MREs, produced small structures of polymeric matrix and particles, and consequently increased the stiffness of MREs. On the other hand, a slight decrease in loss modulus at high magnetic field strength and low frequency was also observed. At this low test frequency, the shear velocity is very low and the particle/matrix interface friction is static friction. With the increase in frequency, particle/matrix interface friction transited from static friction to dynamic friction. This transition caused a decrease in loss modulus and this phenomenon is more obvious at a higher magnetic field [91]. 

**Figure 14:**
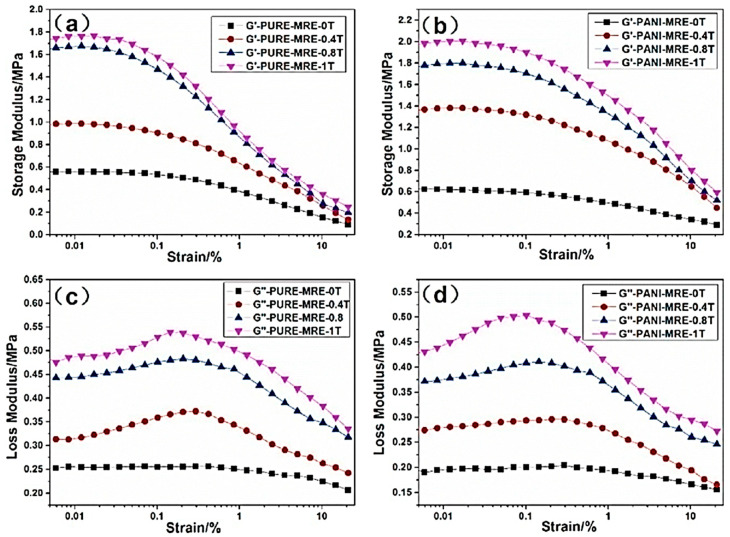
The plot of storage modulus: (**a**) pure MREs, (**b**) PANI-modified MREs, and loss modulus; (**c**) pure MREs and (**d**) PANI-modified MREs vs. strain under various magnetic flux densities [91] (reprinted with permission from Elsevier^TM^).

The storage modulus and loss modulus values of pure and PANI-modified MREs as a function of strain amplitude ranging from 0.005–20% at a fixed frequency of 10 Hz and stationary magnetic flux densities are illustrated in Figure 14. A decrease in the storage modulus was observed with an increase in strain amplitude for all samples (Figure 14a,b). The storage modulus values of PANI-modified MREs were greater than pure MREs at all strain amplitudes. This increase in storage modulus is attributed to the two kinds of particle/matrix and particle/particle interactions. These interactions are highly dependent on the properties of CIPs and matrix interfaces, and the applied magnetic fields. It has been reported that during fabrication, adsorption of polymeric chains occurs on the surface of magnetic particles and develops a core/shell structure in the MRE. The clusters of particles form the core, whereas immobilized polymeric chains form the shell. This core/shell structure considerably participates in improving the rheological properties of MREs [132,133]. On the other hand, the loss modulus of all samples also improved with increasing strain amplitude at a small range of strain, as illustrated in Figure 14c,d. This is because increasing strain amplitude increases the energy required for the continuous rupture of magnetic coupling [134]. With the further increase in strain amplitude, the Payne effect rapidly decreased the loss modulus attributed to the decreased particle interactions and increased particle distances [91].

### 3.8. Viscosity

**Figure 15:**
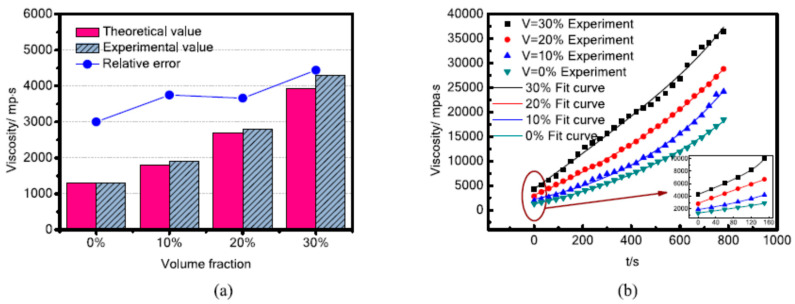
(**a**) The plot of theoretical and experimental values of initial viscosity of MREs containing different volume fractions of magnetic particles. (**b**) Curves of MREs’ viscosity measured during curing [45] (reprinted with permission from Elsevier^TM^).

Previously, the viscosity of MREs suspension was considered constant and independent of time [135]. To explore the effect of curing time on the viscosity of MREs’ suspension, SR-based MREs with varying fractions of CIPs ranging from 0–30 vol% were prepared. Figure 15 illustrated the theoretical and experimental values of the viscosity of MREs calculated through the Einstein–Guth–Gold equation [136], under various curing times and CIP fractions. It was found that the pure SR without CIPs exhibited an experimental viscosity of 1300 MPa. Both the theoretical and experimental viscosities of MREs increased with the increase of CIP fractions (Figure 15a). This is because increasing the fraction of CIPs in MRE considerably improves the adhesion and changes the particle velocity. The change in particle velocity produces a hydrodynamic effect, which causes direct particle/particle interaction and consequently increases the viscosity of MRE suspension [137]. Furthermore, varying curing time also affected the viscosity of MREs suspension, as illustrated in Figure 15b. A double exponential decay function was used to fit the relationship between the viscosities and curing time, as illustrated in Figure 15b [45].

### 3.9. Complex Torsional Stiffness

Zero field stiffness of CIP-filled MREs was higher than the unfilled elastomer, but the application of the magnetic field improved the particle/particle interaction which further improves the stiffness. The complex stiffness values of SR-based MREs containing CIPs were observed to increase with an increase in magnetic field explainable with MR effect. An identical trend was reported in the complex stiffness values of MREs measured under translatory shear conditions [138]. The complex stiffness under 10 Hz and 0 A conditions was 5.194 N-mrad^−1^, whereas the increasing magnetic field to 5 A caused an 8.87% increment in the complex stiffness (5.65 N-mrad^−1^) similar to other frequencies. This increment in complex stiffness is related to the increased particle/particle interactions due to the increased magnetic field [31]. 

**Figure 16:**
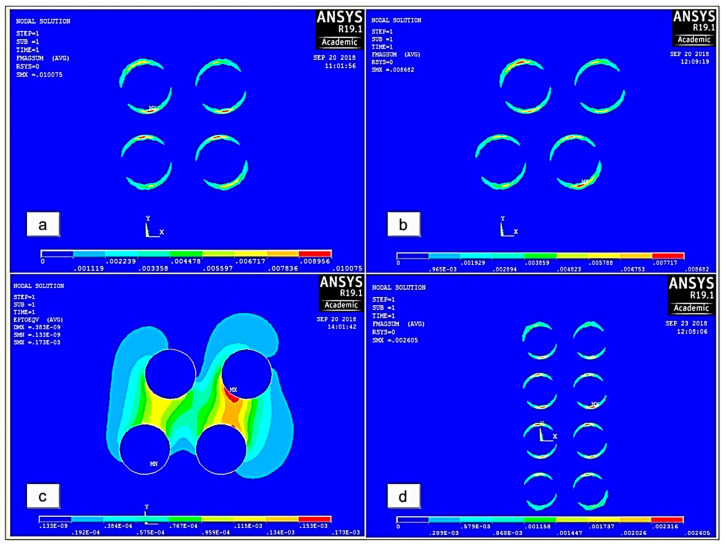
The magnetic force produced on IPs in (**a**) unconstrained and (**b**) constrained conditions; (**c**) mechanical strain on IPs under strained conditions; (**d**) effect of an increase in thickness on the magnetic force of particles [31] (reprinted with permission from Elsevier^TM^).

The results of the simulation of a unit cell performed on ANSYS software in both unconstrained and constrained conditions are illustrated in Figure 16. It was observed that the applied magnetic field aligned the CIPs and produced the magnetic flux, which further developed an attraction force among the CIPs in an unconstrained MRE position (Figure 16a). External torque assigned a new position to CIPs, increased the distances between the dipoles, and consequently reduced the magnetic force on CIPs (Figure 16b). The produced magnetic force of the CIPs exerts a compressive force on the elastomeric matrix which increases the localized complex stiffness of MRE. A mechanical strain of the CIPs as illustrated in Figure 16c presented a compressive force on the SR matrix by CIPs. The effect of thickness of the MRE on magnetic field intensity caused a maximum variation of 8.87% in the MR effect. It was also observed that for fixed sample/dipole distance, the magnetic force of the CIPs decreased with the increased sample thickness, as illustrated in Figure 16d, and signified the reduction in CIPs displacement compared to thinner samples (Figure 16c) [31].

**Figure 17:**
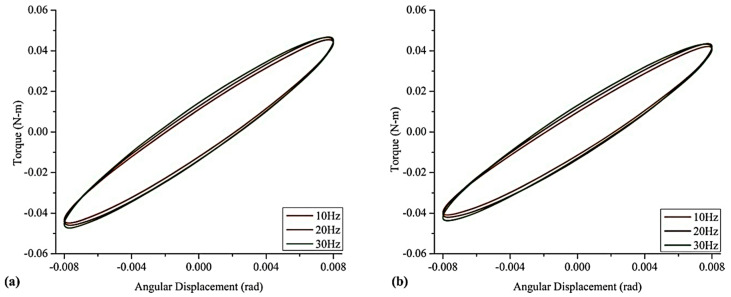
Complex torsional stiffness values at (**a**) 0 A (0 T) and (**b**) 5 A (0.28 T) under various frequencies [31] (reprinted with permission from Elsevier^TM^).

The Lissajous curves of angular displacement and blocked torque for various frequencies ranging from 10–30 Hz at 0 A and 5 A are plotted in Figure 17. Increasing frequency from 10 to 30 Hz caused a smaller shift in the slope. Under the condition of 0 A and 10 Hz, the MRE experienced a maximum torque of 0.0409 Nm, whereas an increase in frequency to 30 Hz increased the torque to 0.0436 Nm. This is associated with the properties sensitive to the frequency of viscoelastic material under the conditions of dynamic loading [139]. Lower frequencies allow polymeric chain molecules to regain their original position but higher frequencies do not. Consequently, elastic material dominates over the viscous material. Without a magnetic field, a maximum 4.68% improvement in the complex stiffness was observed under varying frequencies, whereas the application of a 5 A magnetic field caused a 3.82% improvement. Moreover, frequency-induced improvement of complex stiffness is comparatively less than the magnetic-field-induced improvement [31].

### 3.10. Frictional Properties

**Figure 18:**
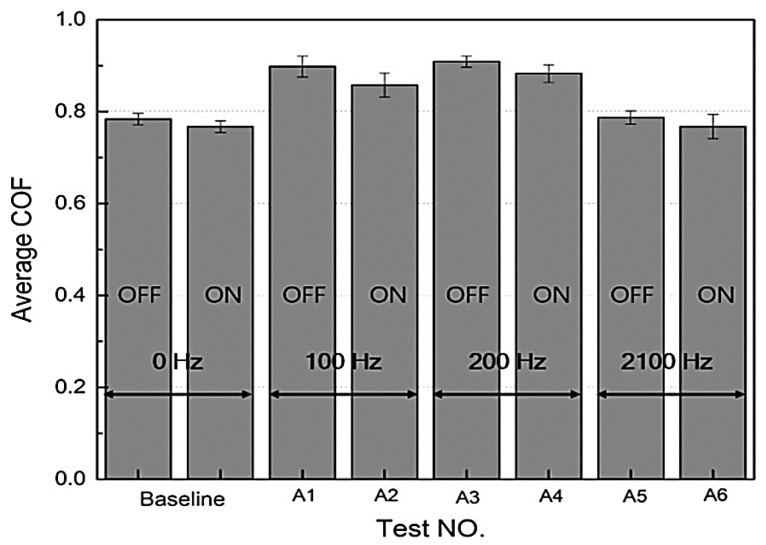
The Plot of the average coefficient of friction as a function of various vibration frequencies [34] (reprinted with permission from Elsevier^TM^).

It has been reported that the values of coefficient of friction of SR- and CIP-based MREs at various frequencies of vibration with magnetic field were lower than without magnetic field and the behavior remained the same at zero vibration. The application of the magnetic field increases the surface hardness of MREs, which leads to very little surface deformation and consequently decreases the coefficient of friction [140,141], regardless of the applied vibration. Under the application of vibration, the coefficient of friction increased except at the resonant frequency. This is attributed to the increased surface temperature, from 27.3 to 29.6 °C, of the MRE due to vibration. The increasing frequency of vibration (100–2100 Hz) increased contact time between the pin and MRE surface and thus increased the coefficient of friction, as illustrated in Figure 18. At the resonant frequency, MRE exhibited a lower value of the coefficient of friction than at 0, 100, and 200 Hz frequencies, which contradicts the literature. This opposite behavior might be due to a momentary reduction in the vertical load of the test system at the resonant frequency. The variations in the coefficient of friction were observed to be lower at a vibration amplitude of 4 mm than at other vibration amplitudes under both with and without magnetic field conditions. This is because of the increased separation between the pin and the MRE surface and the increased acceleration of vibration, resulting in decreased real contact area, momentary vertical load, and normal force [142]. Consequently, the coefficient of friction decreased with increasing vibration amplitude [34]. 

### 3.11. Wear Properties

**Figure 19:**
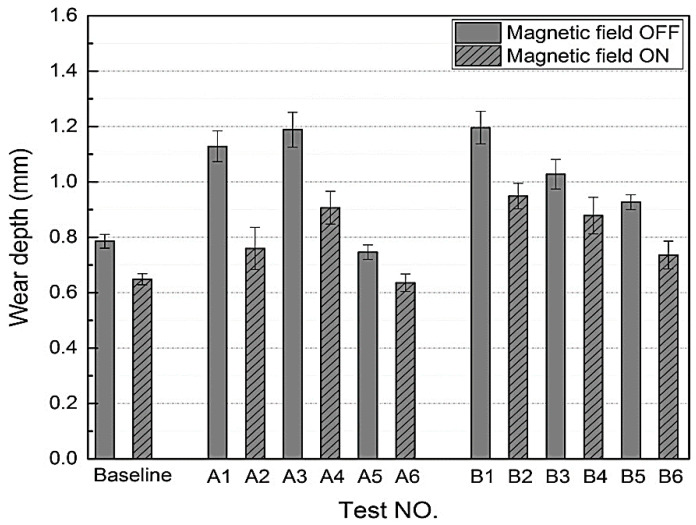
Wear depth obtained after all tests [34] (reprinted with permission from Elsevier^TM^).

The wear test results of SR- and CIP-based MREs are plotted in Figure 19. In the case of baseline, low wear depth and small Schallamach wave were observed with a magnetic field, which can be attributed to the high surface hardness of the MRE. Vibration caused a significant increase in the wear depth and Schallamach wave’s size due to readily material flow at moderate pressures and temperatures. Under constant load and velocity, the wear mainly occurred due to increased vibration, which increased the surface temperature. After the application of the magnetic field, the wear depth and Schallamach wave’s size decreased both with and without vibration conditions. This is because the applied magnetic field increased the surface hardness of MRE, reduced the surface deformation and temperature, and thus reduced the wear depth. The increasing frequency of vibration increased the wear depth and Schallamach wave’s size by increasing the pin and MRE contact time. Furthermore, the increasing amplitude of vibration decreased the wear depth and reduced the Schallamach wave’s size by increasing the distance between two surfaces, increasing the acceleration of vibration, and reducing the real contact area [34].

### 3.12. Fatigue Life of MREs

**Figure 20:**
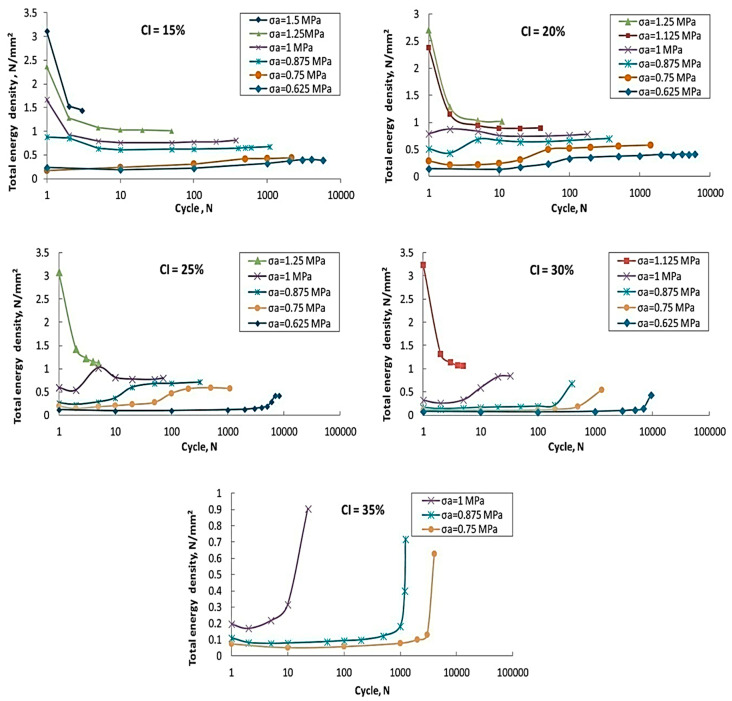
Plots of total energy density vs. cycles under different concentrations of CIPs [35] (reprinted with permission from Elsevier^TM^).

The equi-biaxial fatigue life of SR- and CIP-based MREs were determined by the bubble inflation method. Figure 20 shows the plots of total energy density vs. cycles under various concentrations of CIPs. Variations in total energy density were observed to be highly sensitive to CIPs concentration and stress amplitude. For MREs with CIPs content ranging from 15% to 30%, a decrease in total energy density was observed with an increase in cycles at higher stress amplitudes, whereas an improvement in total energy density was achieved with an increase in cycles at lower stress amplitudes. Irrespective of the applied stress amplitude, total energy density improved with an increase in cycles in the case of MRE with 35% CIP content. A decrease in total energy density at failure was observed when drawn against log10 cycles. This fact suggested that the total energy could be used to predict the fatigue life of MREs subjected to equi-biaxial loading [35]. Uniaxial and biaxial cyclic fatigue properties of NR- and IP-based MREs were investigated between constant strain limits. The uniaxial cyclic fatigue test demonstrated that MREs exhibited stabilized properties at the latter stages of cyclic tests and any change in their properties was attributed to the applied magnetic flux density. An increase in the modulus from 1.325 MPa to 1.413 MPa was observed at the 360th cycle and at strain amplitudes ranging from 0.04–0.08. This was an approximate increase of 6.5% in the 50-cycle block average modulus attributed to the magnetic field applied at the 360th cycle. On the other hand, the block average modulus increased from 3.562 MPa to 3.591 MPa at the 350th cycle and at strain amplitudes ranging from 0.04–0.57. This was an approximate 0.8% increase in mean modulus of the 50-cycle block associated with the applied magnetic field at the 350th cycle. After comparison, it was concluded that increased strain amplitude decreases the MR effect attributed to the increase in separation distance between the particles and reduction in screening effects, whereas the biaxial bubble inflation cyclic fatigue test performed at a low strain of 0.0–0.1 and magnetic flux density of 198 mT showed that an increment in modulus from 3.066 MPa to 3.132 MPa was observed at the 90th cycle. This increment is attributed to the 2.2% improvement in block mean modulus under a specific magnetic flux density. Under the application of relatively higher strain (0–0.5) and the same magnetic flux density, an increment of 1% was observed at the 90th cycle in the block average modulus from 3.375 MPa to 3.410 MPa. In the case of strain amplitude ranging from 0.4–0.5 and the same magnetic flux density, an increment of 0.4% was observed in the MR effect at the 90th cycle from 3.553 MPa to 3.570 MPa [84]. 

### 3.13. Degradation of MREs

FTIR spectroscopy was performed after each processing cycle to evaluate the degradation of the TPE matrix. It has been reported that mechanical and thermal stress increase the atomic vibrations and resist the rotation of molecules, thus causing scission of the polymer chain and formation of free radicals. During processing, these radicals recombine with the present oxygen and cause an increased amount of carbonyl and hydroxyl groups in the oxidized TPE [143]. In FTIR spectra, a broad intensity peak appearing at around 3300 cm^−1^ presented the former group [144], whereas the absorption levels, varying at about 1650 cm^−1^, were assigned to the later ones [145]. The minimum-oxygen-containing groups were observed in the original material; however, oxygen-containing groups increased as the TPE was subjected to reprocessing cycles (R0–R3). The yellowness index (YI) measurements were also performed to evaluate the variations in TPEs by reprocessing. The YI values gradually increased and reached a value two times greater than neat TPE after the last processing cycle. The variations in the molecular weight of both neat matrices and MREs of TPE were also explored by gel permeation chromatography (GPC). The number average molar mass ($\bar{\mathrm{Mn}}$) was observed to decrease with several processing cycles for all samples with a little difference. The neat TPE sample demonstrated the ~14% decrease in $\bar{\mathrm{Mn}}$ after the final processing cycle, whereas the TPE-based MRE exhibited only ~9% decreased $\bar{\mathrm{Mn}}$ value. This implies that the incorporation of CIPs significantly reduced the degradation of TPE. $\bar{\mathrm{Mn}}$ was insignificantly increased on the transition to R1; however, it reduced on the transition to R2 and R3 processing cycles, presenting two transition mechanisms. The first mechanism involved linking of polymer chains individually, whereas the second involved scission of the polymer chain into fragments of lower molecular mass. The key process, which defined the degradation of the TPE matrix or MREs, is the diffusion of oxygen. Oxidation occurs more rapidly at the material surface than inside the material [146]. Therefore, the degradation of TPE-based MREs due to CIPs was negligible and solely attributed to TPE matrix degradation [30].

**Figure 21:**
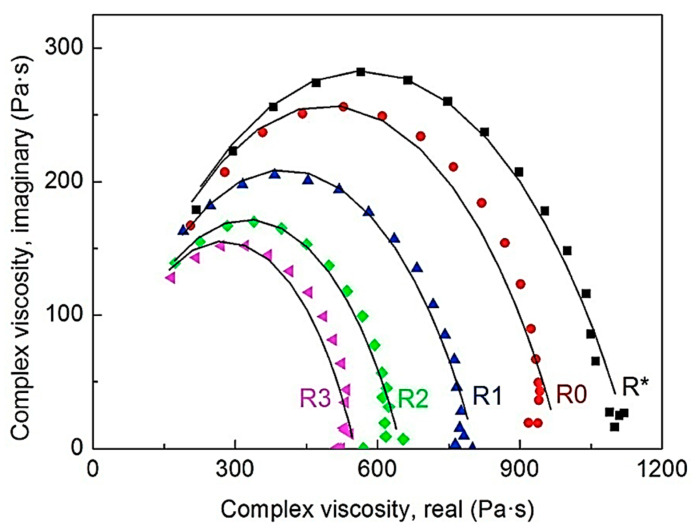
The plot obtained from the Cole–Cole model for original TPE pellets (R*) and neat TPE matrices after each reprocessing cycle (R0–R3). The best model fits are presented by solid lines, and the materials are processed through the injection molding (IM) technique directly by R* [30] (reprinted with permission from Elsevier^TM^).

The processing of polymers can change molecular weight distribution, form low molecular weight fractions, and cause cross-linking of molecules [147]. To analyze the molecular level variations in the TPE matrix during processing, melt rheology was performed. In melt rheology, the TPE melt was subjected to oscillatory shear stress. Due to the viscoelastic nature of polymer melts, the stress, as well as strain, are not in phase [148]. Complex viscosity (η*) is used to describe such a response. Figure 21 illustrated the empirical Cole–Cole model applied to process the complex data. Results showed that the reprocessing cycle number significantly affect TPE properties. Melt rheology proved that the thermomechanical degradation of neat TPE occurred by chain-scission mechanism, whereas this was by recombination processes during reprocessing for MREs. It was also proved that the incorporation of CIPs in MREs strongly affects the degradation of MREs [30].

### 3.14. Capacitance of hybrid-MRE(hMRE)-Based Capacitor

**Figure 22:**
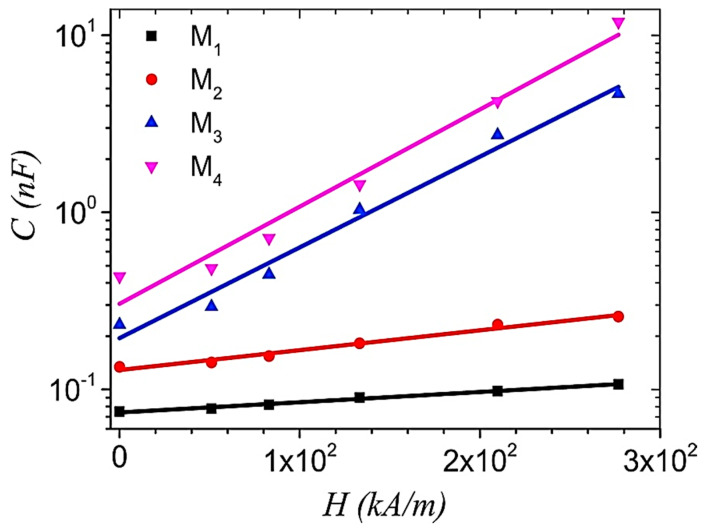
Plot of capacitance (C) of hMRE membrane Mi-based (i = 1, 2, 3, 4) parallel plane capacitor as a function of magnetic field intensity (H) [97] (reprinted with permission from Elsevier^TM^).

Membranes of hybrid MRE consisted of SR, SO, CIPs, and graphene nanoparticles coated with textolite cotton fabric that has been used as a dielectric between the parallel copper plates of a plane capacitor. It has been reported that the increasing magnetic field intensity (H) increased the capacitance of the plane capacitor, as illustrated in Figure 22. The volume concentration of graphene nanoparticles has a significant impact on the capacitance of the plane capacitor. Conversely, the induced mechanical tension in the membrane was observed to be independent of the volume concentration of graphene nanoparticles and increased proportionally to H^2^. The components of deformation were also observed to have a direct relation with H and the volume concentration of graphene nanoparticles. The graphene nanoparticles in the membrane increased the modulus of elasticity in the Hooke model with increasing H and decreased with an increasing volume concentration of graphene nanoparticles [97]. 

## 4. Modern Applications of Isotropic Magnetorheological Elastomers

### 4.1. Sensors and Electrical Circuits

Normally, MREs are electrical insulators, but the addition of graphite powders makes them conductors. The electroconductive MREs, incorporating iron and graphite microparticles in silicon rubber matrix, are used to design active devices of the electric circuit. Several researchers have been working in this field. Ioan Bica [149] developed an MRE-based magnetoresistor sensor device (MRD) and found that the developed MRD satisfactorily fulfilled the function of the active element of an electric circuit or/and quadrupolar magnetoresistor. The output voltage of the developed MRD was observed to increase with the increase in magnetic field intensity and control voltages. Similarly, Bica et al. [150] worked on the effect of the magnetic field and compression pressure on the electrical conductivity of hybrid electroconductive MREs, incorporating graphene nanoparticles and CIPs in the silicon rubber matrix. They reported that the electrical conductivity of fabricated hybrid MREs increased with an increase in compression pressure and magnetic field intensity. Electroconductive MREs can also be used as dielectric material in electrical condensers. The capacity of such MRE-based condensers can be altered by applying an external magnetic field, associated with the magnetostrictive effect. Due to this property, electroconductive MREs are widely used in devising thermosensors, Hall sensors, and magnetoresistors [151].

### 4.2. Soft Robotics

Various investigations are in progress in the field of soft robotics on the modeling, manufacturing, design, and control of soft robots [152]. Reduction of soft robotic tethering or the dependence of soft robot on a base station for pressure, power, and other forms of regulation is still a challenge [153]. The fields of biomimicry, robotic grasping, and biomedicine are taking interest in soft robotics using soft actuators [154,155]. To achieve guidance and manipulation of soft actuators, MREs with both soft and hard magnetic filler particles are the most suitable materials for several applications. Hybrid MREs with varying magnetic domains have been utilized in creating soft robotic swimmers. Similarly, MRE-based millimeter-scale soft robots with the abilities of swimming, rotating, and rolling with varying external magnetic field intensities and directions have also been produced. The use of MREs enabled the addition of a triangular tail and controlling the undulating gait behavior of soft robotics. A thin, long, MRE-based microbot was produced for biomedical applications, which can be guided remotely within a 3D phantom vascular network. An MRE-based, wearable, magnetic skin has also been developed for various applications, such as remote gesture and eye-tracking control during coordination with other sensors [156].

## 5. Conclusions and Outlook

This review paper outlines the recent research on isotropic MREs and their properties, developed in the last five years. The isotropic MREs fabrication parameters are rigorously discussed, including preparation of magnetic filler particles; types of matrices, i.e., liquid silicone, RTV-SR, HTV-SR, EPDM, PUR, PDMS rubber, propylene rubber, SR resin, NR, and scrub tire rubber; types of magnetic filler particles, such as BIPs, CIPs, Penta CIPs, Fe_3_O_4_, TiO_2_, and hard FeNdB particles; magnetic filler particles sizes, shapes, and volume fractions; types of additives, curing time/temperatures, and fabrication method. The properties of isotropic MREs, such as morphological, mechanical, magneto-mechanical, magneto-shear, rheological, melt rheological, tomographical, friction, and wear properties, as well as complex torsional stiffness and fatigue life have been reviewed in detail. The isotropic MREs have been extensively used in various applications, such as automotive, medical, robotics, electric and electronic devices, sensors, polishing composites, seismic dampers, and base isolators for many years. But in the last few years, efforts have been more devoted to the development of modern MREs with superior properties and the improvement of properties of existing isotropic MREs. However, the evaluation and improvement of friction, wear, and thermal properties of isotropic MREs have not been given much attention. A blend of various rubbers should be used as a matrix in isotropic MREs, having the combined properties of all rubbers in the blend. The effect of Gamma radiation on the properties of different isotropic MREs still needs to be explored.

## Figures and Tables

**Table 1:** Elastomers, filler particles, additives, and key parameters used for the fabrication of isotropic MREs. PDMS—polydimethylsiloxane; PUR—polyurethane; SR—silicone rubber; EPDM—ethylene propylene diene rubber.

Matrix	Filler Particles	Particles Size (µm)	Particles Content	Additives	Curing Temp (°C)	Curing Time (min)	Ref.
SR	CIPs	2–5	27 vol%	Catalyst	RT	1440	[73]
SR	CIPs	5	27 vol%	Silicone oil	RT	1440	[31]
SR	CIPs	3–5	0–30 vol%	PDMS	25	10	[45]
SR	CIPs	3.9–5	30 vol%	–	RT	1440	[19]
SR	CIPs	3.9–5	12.5–40 vol%	Slacker, Silicone thinner	65	20	[95]
SR	CIPs	3.9–5	5–40 vol%	–	RT	1440	[83]
SR	CIPs	4.5–5.4	20 vol%	Silicone oil, Graphene nano powder,	RT		[97]
SR	CIPs	5.89	20 vol%	Silicone oil	60	120	[29]
SR	CIPs	6–7	15–35 vol%	Catalyst	RT	2880	[35]
SR	CIPs	5–9	10–40 wt%	Dimethyl silicone oil,	RT	720	[23]
SR	CIPs	1−10	70 wt%	Ethanol, Curing Agent	RT	120	[78]
SR	CIPs	40	40 wt%	1,3-divinyl-1,1,3-Tetramethyldisiloxane	–	–	[22]
SR Resin	CIPs	3–5	70 wt%	–	25	1440	[89]
NR	IPs	–	18.3 vol%	Carbon Black	–	–	[90]
NR	IPs	–	18.3 vol%	Carbon Black	–	–	[84]
NR	CIPs	1.25	0–30 vol%	–	180	10	[27]
PUR	CIPs	4.9	–	Silicone oil	–	–	[12]
SR, PUR	TiO_2_, CIPs	5	10 vol%	–	–	–	[61]
PUR	CIPs	1–8	60 wt%	Aniline, Ammonium, Peroxodisulfate, p-toluenesulfonic Acid, 2,4,6-Tri(dimethylaminomethyl)phenol, Di-butyl phthalate,	–	–	[91]
PUR	CIPs	6–9	33 vol%	–	–	–	[98]
Propylene- Rub	CIPs	6.5–8	31 vol%	–	–	–	[30]
EPDM	CIPs, IPs	5−16	2–30 phr	Carbon Black, Sulfur, Processing oil, Activators, Antidegradants, Accelerators	180	10	[24]
PDMS	IPs	20–80	7.66 vol%	–	RT	–	[77]
Waste Tire Rubber	Penta-CIPs	6	10–40 wt%	Carbon black, Rubber additives, Minerals, Sulfur	200	60	[92]
Scrub Tire Rub	IPs	16.99	10–40 wt%	–	200	17–20	[32]
Scrap Tire Rub	Magnetite (Fe_3_O_4_)	–	10–40 wt%	–	200	17–20	[33]
